# The complete mitochondrial genome of *Tetragonia tetragonioides* (Pall.) Kuntze and its phylogenetic implications

**DOI:** 10.1080/23802359.2021.1967805

**Published:** 2021-08-27

**Authors:** Shunxin Hu, Jingxi Liu, Lizhu Chen, Wei Chen, Yanxiang Li, Jiqing Gao, Rao Fu, Chunxiao Sun, Bin Li, Junwei Tang, Peng Qiao, Meng Li, Zhijie Ni, Mingmei Hao

**Affiliations:** a Shandong Provincial Key laboratory of Marine Ecological Restoration, Shandong Marine Resource and Environment Research Institute, Yantai, China; b Changdao National Marine Park Management Center, Yantai, China; c Shandong Institute of Sericulture, Yantai, China; d Changdao Marine Economy Promotion Center, Yantai, China; e National Demonstration Center for Experimental Fisheries Science Education, Shanghai Ocean University, Shanghai, China

**Keywords:** Mitochondrial genome, *Tetragonia tetragonioides*, phylogenetic, implications, Aizoaceae

## Abstract

The complete mitochondrial genome sequence of *Tetragonia tetragonioides* (Pall.) Kuntze was assembled and characterized in the present study. The mitochondrial genome was 347,227 bp in length and had a GC content of 43.84%, including 24 transfer RNA (tRNA) genes and three ribosomal RNA (rRNA) genes. Phylogenetic analysis showed that *T. tetragonioides* was close to and *Sesuvium portulacastrum*.

The *Tetragonia tetragonioides* (Pall.) Kuntze is an annual plant belonging to the family Aizoaceae. It is widely distributed in subtropical and temperate coastal salt-rich areas, which is rich in iron, calcium and a variety of vitamins. It can also be used as medicine to clear heat and detoxify, dispel wind and detumescence, implying a high medicinal value. As a kind of halophyte with high salinity tolerance, the *T. tetragonioides* can tolerate seawater and survive normally on salinized land. It can be used for the improvement and utilization of seawater irrigation farmland in salt wasteland of coastal land (Wilson et al. 2000).

Fresh leave samples were collected from *T. tetragonioides* in Binzhou, China (N 38.20, E 117.96). The samples were deposited at the Shandong Marine Resources and Environment Research Institute (number: SMRERI_20200712, collected by Shunxin Hu, e-mail: hushunxin001@163.com). Genomic DNA was extracted from the samples with Qiagen DNeasy Plant Mini Kit (Qiagen, Carlsbad, CA, USA). The mitochondrial genome of *T. tetragonioides* was sequenced using a combination of the Illumina NovaSeq 6000 platform and PacBio Sequel II. We used the GetOrganelle v1.7.1 to assemble the Illumina sequencing data. The assembled sequences were checked whether there are overlapping sequences, which were then manually corrected.

The average read coverage of the genome was 3209. Finally, the complete mitochondrial genome of *T. tetragonioides* was a circular form of 347,227 bp, which had a GC content of 43.84%. The total length of the protein-coding genes was 29,229 bp, accounting for 8.42% of the genome length. The total length of non-coding-proteins genes was 4658 bp, including 24 transfer RNA (tRNA) genes and three ribosomal RNA (rRNA) genes (*rrn*18, *rrn*5, *rrn*26). In addition, we totally found 22 introns in nine genes (*nad*1, *nad*2, *nad*4, *nad*5, *nad*7, *ccmFc*, *rps*3, *clpP*1, *cox*2).

In order to further investigate the phylogenetic position of *T. tetragonioides*, we construct a phylogenetic analysis with the mitochondrial genomes of other 13 plants. The mitochondrial genomes sequence of *Ginkgo biloba* were used as an outgroup (Figure 1). The 14 mitochondrial genomes were aligned by MAFFT v7.307 (Katoh and Standley 2013). And maximum likelihood (ML) tree was performed by Mega 7.0 (Sudhir et al. 2016). The GTR + G model was selected for ML analyses with 1000 bootstrap replicates. And the ML tree was visualized by using iTOL v3.4.3 (Letunic and Bork 2016). The phylogenetic results showed that the *T. tetragonioides* was close to *Sesuvium portulacastrum* (Figure 1), since they were both in the Aizoaceae. And the mitochondrial genome information of *T. tetragonioides* will provide data for further analysis of evolutionary history.

**Figure 1.**
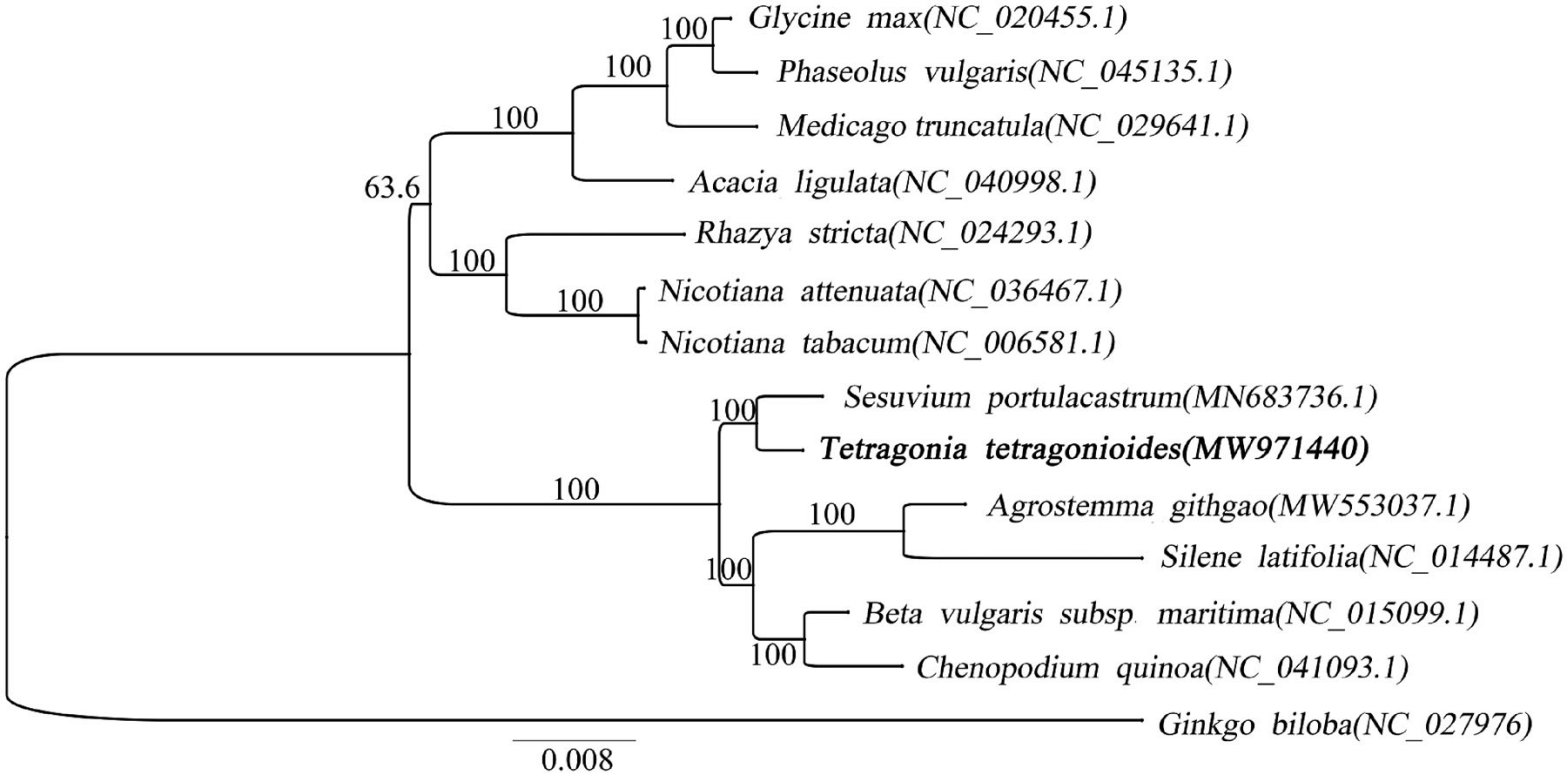
ML phylogenetic tree of the Brassicaceae based on the 13 mitochondrion genome sequences in GenBank, plus the mitochondrion sequence of *Tetragonia tetragonioides*. The *Ginkgo biloba* was used as outgroup with the bootstraps (1000 replicates).

## Data Availability

The genome sequence data that support the findings of this study are openly available in GenBank of NCBI at (https://www.ncbi.nlm.nih.gov/) under the accession no. MW971440. The associated BioProject, SRA, and Bio-Sample numbers are PRJNA740354, SRP325516, and SAMN19842629 respectively.

## References

[CIT0001] Katoh K, Standley DM. 2013. MAFFT multiple sequence alignment software version 7: improvements in performance and usability. Mol Biol Evol. 30(4):772–780. 23329690 10.1093/molbev/mst010 PMC3603318

[CIT0002] Letunic I, Bork P. 2016. Interactive tree of life (iTOL) v3: an online tool for the display and annotation of phylogenetic and other trees. Nucleic Acids Res. 44(W1):W242–W245. 27095192 10.1093/nar/gkw290 PMC4987883

[CIT0003] Sudhir K, Glen S, Koichiro T. 2016. Mega7: molecular evolutionary genetics analysis version 7.0 for bigger datasets. Mol Biol Evol. 33(7):1870–1874. 27004904 10.1093/molbev/msw054 PMC8210823

[CIT0004] Wilson C, Lesch SM, Grieve CM. 2000. Growth stage modulates salinity tolerance of New Zealand spinach (*Tetragonia tetragonioides*, Pall.) and red orach (Atriplex hortensis L.). Ann Bot-London. 85(4):501–509.

